# Flipped classroom integrated with team-based learning enhances surgical skills in ophthalmology residency training: a randomized controlled trial

**DOI:** 10.3389/fmed.2026.1859841

**Published:** 2026-06-04

**Authors:** Zhuoqiong Wang, Ji Xu, Xinxin Hu, Weina Ren, Shuanghua Xin, Na Zhao, Luyao Ye, Qinkang Lu, Juntao Zhang

**Affiliations:** 1 Department of Ophthalmology, The Affiliated People's Hospital of Ningbo University, Ningbo, Zhejiang, China; 2 Department of Ultrasound Medicine, The Affiliated People's Hospital of Ningbo University, Ningbo, Zhejiang, China

**Keywords:** flipped classroom, ophthalmology residency training, randomized controlled trial, surgical skills, team-based learning

## Abstract

**Background:**

Standardized ophthalmology residency training emphasizes competency-based education, with fine surgical skills representing a key challenge. However, the current traditional lecture-based teaching program has a long learning curve due to insufficient active participation and practice time. The flipped classroom combined with team-based learning (TBL) may address this gap. Therefore, this study aimed to evaluate the application value of this novel teaching method in surgical skills training for ophthalmology residents.

**Methods:**

Twenty residents who underwent standardized ophthalmology training at the Affiliated People's Hospital of Ningbo University from January 2026 to March 2026 were enrolled and randomly divided into an experimental group (*n* = 10) and a control group (*n* = 10). The experimental group received flipped classroom combined with TBL, while the control group received traditional lecture-based teaching. Training was conducted in a wet lab. Theoretical examinations were administered before and after training, practical skill assessments were conducted after training, and a teaching satisfaction questionnaire was administered.

**Results:**

There were no significant differences in pre-training theoretical scores between the two groups (*p* > 0.05). After training, the theoretical performance of the experimental group was significantly higher than that of the control group (*p* < 0.05, Cohen's *d* = 0.95). In the post-training skill assessment, the operation process score (*p* < 0.001, Cohen's *d* = 4.43) and total score (*p* < 0.001, *d* = 5.29) of the experimental group were significantly better than those of the control group. In other four modules of pre-operation preparation, post-operation processing, operation proficiency, and question answering, the experimental group was also better than the control group (*p* < 0.05). The questionnaire survey showed that the satisfaction level of the experimental group in the three dimensions of “improvement of operational ability”, “enthusiasm of teachers” and “overall self-evaluation” was higher than that of the control group (*p* < 0.05). There was no significant difference between the two groups in the remaining dimensions (*p* > 0.05).

**Conclusions:**

This study confirms that the flipped classroom combined with TBL teaching method can significantly improve the performance of ophthalmic residency skills training, and its teaching effect is significantly better than that of the traditional model. It is an innovative teaching mode with high application value in ophthalmology surgical skills training.

## 1 Introduction

The core of standardized training for ophthalmology residents in China is to cultivate talents with job competency (1). This requires residents to develop not only a systematic clinical diagnostic mindset but also a high level of operational skills, ranging from basic examinations to complex surgical procedures. Ophthalmic surgery involves fine anatomical structures and demands high surgical precision (2). Mastering these skills requires solid theoretical knowledge, repeated hands-on practice, precise hand-eye coordination, and timely, effective constructive feedback (3). Therefore, skill manipulation remains a daunting and critical challenge in the training content of ophthalmology residents.

Currently, ophthalmology surgical skills training still relies on the traditional method of teacher-led instruction with passive student absorption. This approach is relatively rigid in format, and the lecture-based delivery of theoretical knowledge yields only modest teaching outcomes, making it difficult to engage students' initiative (4). Moreover, lengthy lectures on theoretical content consume considerable time, which significantly reduces the time available for hands-on practice. Consequently, each student has fewer opportunities for actual operative practice, thereby prolonging the learning curve. Therefore, traditional teaching methods are characterized by a long learning cycle and low learning efficiency in surgical skills training, rendering them insufficient to meet the growing demand for high-quality talents (5).

The application of the flipped classroom teaching method has become widespread in higher education (6). Compared with traditional teaching, flipped classrooms offer significant advantages in improving learning effectiveness, participation, and student satisfaction (7–16). This approach shifts the knowledge input phase to before class, where students independently learn theoretical content through materials provided by the instructor. Knowledge internalization then occurs in the classroom through hands-on practice, discussions, assessments, and other interactive activities (17). This restructured teaching process effectively increases students' hands-on practice time in class, enhances classroom learning efficiency, and fosters active learning motivation, thereby addressing the limitations of traditional teaching methods in surgical skills training (18).

Ophthalmic surgical procedures are generally more delicate and complex. In addition to requiring substantial practice time, students must actively exercise their own initiative. Therefore, transforming the passive learning mode in surgical skills teaching is particularly important. Team-based learning (TBL) emphasizes highly structured and interactive instruction, with a focus on improving students' individual problem-solving skills (19). Compared with traditional teaching methods, the application of TBL has been shown to help students effectively improve their post-training theoretical performance and enhance collaboration within study groups (20–22). Thus, a small-class team teaching model can meet the needs of ophthalmic surgical skills training by stimulating students' intrinsic motivation and promoting deep learning and skill mastery.

However, research on the combined application of flipped classroom and TBL in ophthalmic surgical training is currently lacking. Therefore, this study aimed to further evaluate the application value of the flipped classroom combined with TBL method in surgical skills training for ophthalmology residents by comparing the training outcomes between a traditional teaching group and a group receiving the novel teaching method.

## 2 Materials and methods

### 2.1 Participants

A total of twenty residents who underwent standardized ophthalmology training in the People's Hospital of Ningbo University from January 2026 to March 2026 were selected, and none of them had previously participated in the training related to pterygium resection surgery, and this study has been approved by the Medical Ethics Committee of the People's Hospital Affiliated to Ningbo University (approval no.: 2026 Scientific Research No. 049).

### 2.2 Study design

The pilot experiment (with 5 residents in each group, not included in the formal trial) showed a ≈10-point difference favoring the experimental group (Total score 81.6 ± 2.79 vs. 71.2 ± 2.86, Cohen's *d* ≈ 3.68). According to the sample size calculation formula (superiority test, one-sided α = 0.025, power 1–β = 0.90, superiority margin Δ set to a clinically reasonable value of 5 points—approximately 50% of the observed difference between the two groups), about 6–7 cases per group are needed to achieve 90% power to detect a difference. This study adopted a controlled experimental design. Twenty participants were randomly divided into an experimental group (*n* = 10) and a control group (*n* = 10) using a lottery-based randomization method. The experimental group received flipped classroom combined with TBL, while the control group received traditional lecture-based teaching. Pterygium excision surgery was selected as the teaching content for this educational study. This procedure is not only one of the fundamental ophthalmic surgical skills but also involves numerous fine anatomical structures and operative essentials that students must master, with graded levels of difficulty.

### 2.3 Teaching process

Before the teaching activities began, all participants completed a theoretical examination on pterygium excision surgery, with the results recorded as pre-training theoretical scores to assess their baseline knowledge. Subsequently, group-specific teaching activities were implemented. Skill practice and assessments for both groups were conducted in a wet lab environment, using isolated porcine eyeballs as the surgical training model. To simulate a realistic surgical setting, each porcine eyeball was fixed in a simulated orbital device. Key steps of pterygium excision surgery—including conjunctival incision, pterygium head dissection, body resection, scleral bed cleaning, and conjunctival suturing—were performed under a binocular surgical microscope. The two groups were matched for training content, class duration, surgical instruments, and consumables. All sessions took place in the same wet laboratory, and the teacher was the same senior ophthalmologist for both groups, ensuring that all variables except the teaching method were effectively controlled.

#### 2.3.1 Teaching process in the experimental group

**Figure 1:**
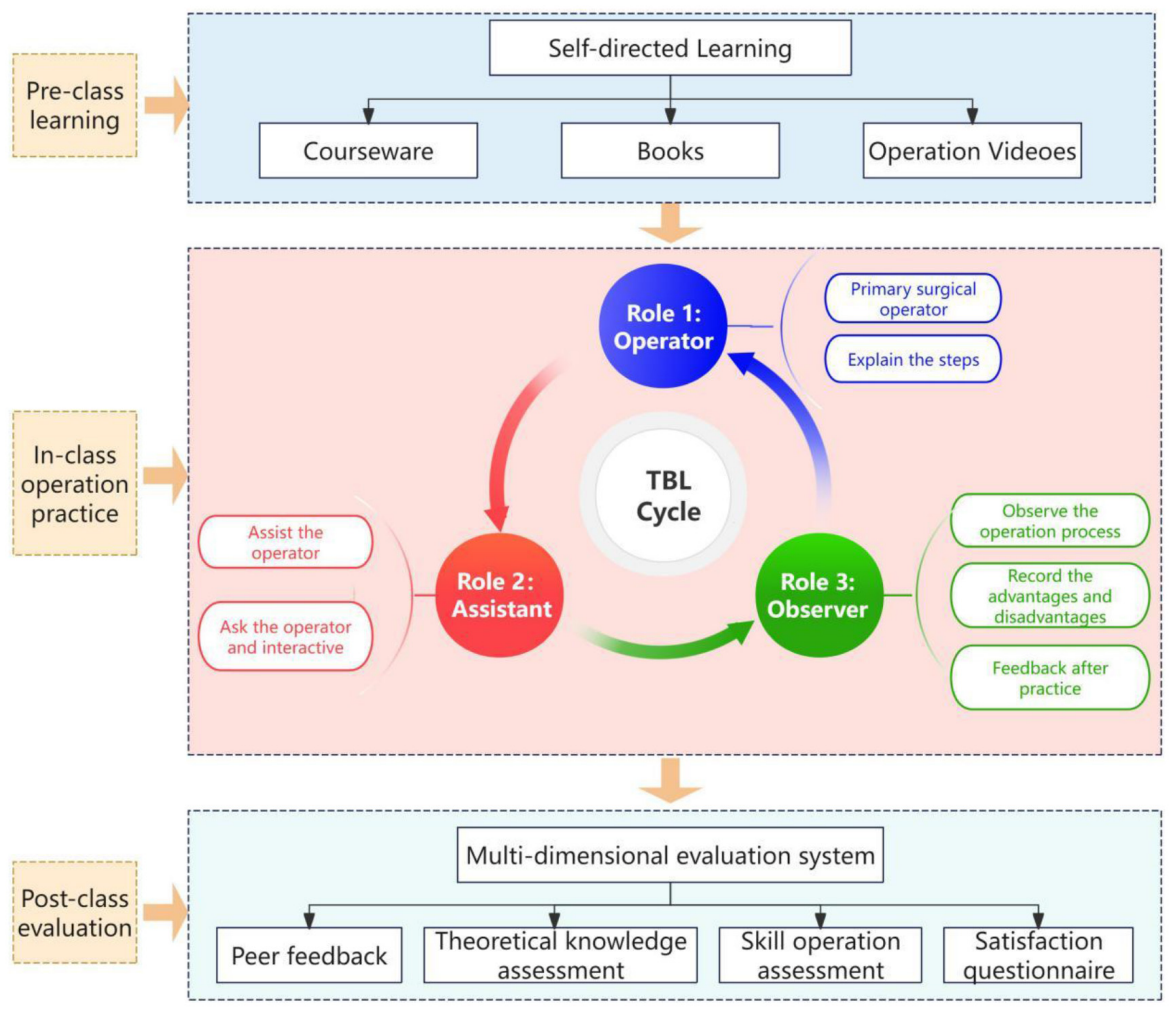
Teaching process of flipped classroom combined with TBL.

1. The ten residents in the experimental group were randomly divided into two TBL groups, with five residents in each subgroup.
2. Pre-class learning: the teacher provided relevant learning materials to the residents in advance, including but not limited to courseware, textbooks, and expert operative videos covering pterygium anatomy, stepwise surgical techniques, and other relevant knowledge. Residents were required to complete independent learning before class. Concurrently, the teacher informed the residents that the pre-class learning objective was for each resident to be able to explain and teach the content during in-class practice sessions.
3. In-class operation practice: under the teacher's supervision, residents performed pterygium excision surgery on animal eyes. During practice, the residents worked in their designated TBL groups, cycling among three roles: operator, surgical assistant, and observer. These roles were designed to ensure both individual accountability, meaning each resident had to master all roles, and group interdependence, meaning that successful completion required active collaboration. The specific responsibilities for each role were as follows: the operator performed the main surgical procedure while simultaneously explaining the operative steps and key precautions; the assistant aided the operator in completing the procedure and posed questions, which the operator was responsible for answering; the observer recorded the strengths and weaknesses of the operator's performance and provided feedback and evaluation after each practice session. In line with TBL's facilitator role for instructors, the teacher provided timely guidance and answered questions when necessary but did not intervene or offer continuous commentary throughout the entire procedure. After each resident had assumed all three roles, structured peer feedback and evaluation were exchanged, and learning experiences were shared—reinforcing TBL's emphasis on team reflection and collaborative knowledge construction.
4. Post-class assessment and evaluation: after training, all residents completed a theoretical examination on the relevant content, with the results recorded as post-training theoretical assessment scores. The scoring criteria were adapted from the Zhejiang Provincial Standardized Residency Training Clinical Practice Ability Completion Assessment and the Pterygium Excision Scoring Table for Ophthalmic Surgery. Learning outcomes were objectively evaluated and recorded using quantitative indicators (Figure 1).

#### 2.3.2 Teaching process in the control group

1. In-class theoretical teaching: the teacher delivered a unified lecture on the theory of pterygium excision surgery. When necessary, the teacher interacted with the residents in a timely manner and answered their questions.
2. In-class operation practice: the teacher first demonstrated pterygium excision surgery on an animal eye, explaining the key steps, techniques, and precautions. Subsequently, the remaining class time was allocated for residents to practice independently, with the teacher providing guidance and corrective feedback as needed.
3. Post-class assessment and evaluation: this process was consistent with that of the experimental group. Residents completed theoretical and practical skill assessments after training, and their scores were recorded.

#### 2.3.3 Questionnaire survey

Based on the “Construction of the Independent Learning Ability Assessment Scale for Medical Students” by Wang XD (23), all participants were invited to complete the questionnaire truthfully. The collected data were recorded for analysis. Each item was scored on a 4-point Likert scale, with the following response options and corresponding scores: 4 points = very satisfied, 3 points = satisfied, 2 points = dissatisfied, and 1 point = very dissatisfied. Higher scores indicated higher levels of satisfaction. The questionnaire consisted of eight items: Do you think this teaching activity helps consolidate your theoretical professional knowledge? Do you think this teaching activity helps improve your skill operation level? Do you think this teaching process has sparked a strong interest in ophthalmic skills operation teaching? Do you think this teaching process did enhance your self-learning ability? Do you think the instructor showed strong teaching enthusiasm during this teaching activity? Do you think this teaching method provides a greater sense of participation compared to previous methods? How satisfied are you with your own performance in this teaching process? What is your overall satisfaction with this teaching activity? Reliability and validity analyses were conducted. The Cronbach's α coefficient was 0.947, indicating excellent internal consistency. The KMO value was 0.749 (>0.7), and Bartlett's test of sphericity was significant (χ^2^ = 240.133, df = 28, *p* < 0.001), confirming the suitability of the data for factor analysis. A single factor with an eigenvalue of 6.62 explained 82.80% of the total variance, well above the recommended 50% threshold. Factor loadings for all eight items ranged from 0.66 to 0.98, all exceeding 0.5. In summary, the questionnaire demonstrated excellent reliability and good construct validity, making it suitable for evaluating the effectiveness of ophthalmic skills training activities.

### 2.4 Data analysis

SPSS 30.0 software was used for statistical analysis. The Shapiro–Wilk test was applied to assess the normality of all continuous data. Variables following a normal distribution were expressed as mean ± standard deviation (mean ± SD), and between-group differences were compared using Welch's *t*-test. Variables not following a normal distribution were expressed as median with interquartile range [M (Q1, Q3)], and between-group differences were compared using the Mann–Whitney U test. A *p*-value < 0.05 was considered statistically significant.

Effect sizes were calculated to reflect the practical magnitude of differences. For normally distributed data, Cohen's d was used. For non-normally distributed data, Cliff's δ was used. According to the criteria proposed by Cohen (24), values of 0.2, 0.5, and 0.8 correspond to small, medium, and large effects, respectively. To enable uniform interpretation of effect sizes, Cliff's δ was converted to Cohen's *d* using the formula recommended by Ruscio (25):


$$\begin{array}{l} d=\frac{2\delta }{\sqrt{1-\delta^{2}}} \end{array}$$

## 3 Results

### 3.1 Baseline characteristics

The mean age of residents in the experimental group and control group was 26.40 ± 1.71 years and 25.50 ± 1.35 years, respectively, with no statistically significant difference (*p* = 0.209). The sex distribution between the two groups also showed no significant difference (*p* = 0.303). In the experimental group, nine residents held a master's degree and one held a bachelor's degree; all ten residents in the control group held a master's degree, with no significant difference between groups (*p* = 1.000). None of the residents in either group had prior learning experience with pterygium excision surgery. Overall, there were no significant differences between the two groups in baseline characteristics such as age, sex, and educational level, indicating that the two groups were comparable.

### 3.2 Comparison of teaching effects

#### 3.2.1 Comparison of theoretical scores between the two groups

The theoretical scores of the two groups before and after training are presented in Table 1. Before training, there was no significant difference between the two groups (*t* = −0.30, *p* > 0.05), indicating similar baseline levels and comparability. After training, the experimental group achieved significantly higher theoretical scores than the control group (*t* = −2.11, *p* < 0.05). The absolute value of Cohen's *d* was 0.95, exceeding the threshold for a large effect (> 0.8 according to Cohen's criteria).

**Table 1:** Comparison of theoretical scores between the two groups before and after training.

Indicator	Experimental group (*n* = 10)	Control group (*n* = 10)	*t*	*p*	Cohen's *d*
Pre-training theoretical score (mean ± SD)	72.00 ± 6.75	71.00 ± 8.10	−0.30	0.768	−0.13
Post-training theoretical score (mean ± SD)	87.50 ± 4.86	81.00 ± 8.43	−2.11	0.049	−0.95

#### 3.2.2 Comparison of post-training skill assessment results between the two groups

The skill assessment results of the two groups after training are detailed in Table 2. The pterygium excision scoring table comprised five sections: pre-operative preparation, operative procedure, post-operative handling, operational proficiency, and question answering. According to the Shapiro–Wilk test, the operative procedure score and the total score followed a normal distribution and were therefore compared using an independent-samples *t*-test. The scores for pre-operative preparation, post-operative handling, operational proficiency, and question answering did not follow a normal distribution and were compared using the Mann–Whitney U test.

**Table 2:** Comparison of skill assessment scores between the two groups after training.

Assessment component	Experimental group (*n* = 10)	Control group (*n* = 10)	Test statistic	*p*	Cohen's *d*
Pre-operative preparation M(Q1, Q3)	13.5 [11.0, 14.5]	10.0[8.0,12.0]	*U*=16.50	0.008	1.50
Operative procedure (mean ± SD)	51.10 ± 2.42	41.90 ± 1.66	*t* = −9.89	< 0.001	4.43
Post-operative handling, M(Q1, Q3)	9.0 [8.0, 10.0]	4.0 [3.0, 5.0]	*U* = 2.50	< 0.001	2.88
Operational proficiency, M(Q1, Q3)	9.0 [8.0, 9.0]	6.0 [5.0, 7.0]	*U* = 5.50	< 0.001	2.44
Question answering, M(Q1, Q3)	5.0 [4.0, 5.0]	3.0 [2.0, 4.0]	*U* = 16.00	0.003	1.76
Total score (mean ± SD)	87.00 ± 3.13	72.10 ± 2.47	*t* = −11.82	< 0.001	5.29

According to the results, the experimental group achieved a significantly higher score than the control group in the operative procedure component (51.10 ± 2.42 vs. 41.90 ± 1.66), with a statistically significant difference (*p* < 0.001) and a large effect size (Cohen's *d* = 4.43). The total skill assessment score was also significantly higher in the experimental group than in the control group (87.00 ± 3.13 vs. 72.10 ± 2.47, *p* < 0.001), with a very large effect size (Cohen's *d* = 5.29).

For the remaining four indicators that did not follow a normal distribution, the experimental group outperformed the control group in all cases. The scores, test statistics, and effect sizes are presented in Table 2. All *p*-values were ≤ 0.05, indicating statistically significant differences. The converted Cohen's *d* values ranged from 1.50 to 2.88, all representing large effects.

**Figure 2:**
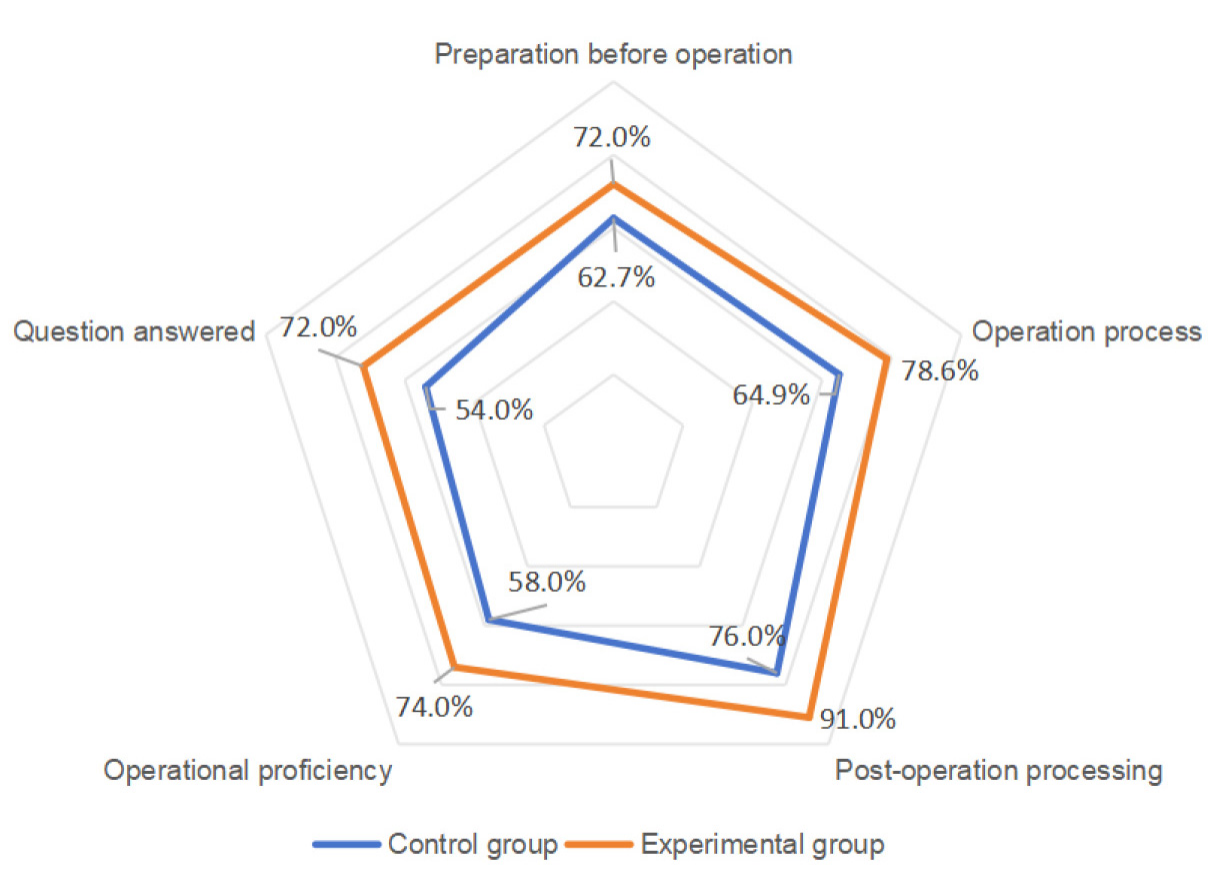
Comparison of the score rate of each module of the skill operation assessment between the two groups.

Furthermore, in the practical skill assessment, the experimental group achieved a higher scoring rate than the control group in each module. The most pronounced differences were observed in post-operative handling (91.0 vs. 76.0%), operative procedure (78.6 vs. 64.9%), and operational proficiency (74.0 vs. 58.0%), detailed in Figure 2.

### 3.3 Questionnaire survey results

The Mann–Whitney U test was used to compare satisfaction differences between the experimental group and the control group across each dimension. Due to the small sample size, exact two-tailed significance values are reported. Scores for each group are presented as median with interquartile range [M (Q1, Q3)], and the results are detailed in Table 3.

**Table 3:** Comparison of questionnaire survey results between the two groups.

Questionnaire content	Experimental group (*n* = 10)	Control group (*n* = 10)	*U*	*Z*	*p*
Improvement in theoretical knowledge	4.0 (4.0,4.0)	3.5 (3.0,4.0)	34.000	−1.446	0.247
Improvement in operational ability	4.0 (4.0,4.0)	3.0 (2.0,4.0)	20.000	−2.809	0.023
Interest in learning	4.0 (4.0,4.0)	3.5 (2.0,4.0)	28.000	−2.063	0.105
Self-study ability	4.0 (4.0,4.0)	2.5 (1.0,4.0)	28.000	−1.851	0.105
Teacher enthusiasm	4.0 (4.0,4.0)	3.0 (3.0,4.0)	15.000	−3.162	0.007
Sense of participation	4.0 (4.0,4.0)	3.5 (3.0,4.0)	25.000	−2.492	0.063
Overall self-evaluation	4.0 (4.0,4.0)	3.0 (2.0,4.0)	18.000	−2.757	0.015
Overall satisfaction	4.0 (4.0,4.0)	3.0 (2.0,4.0)	25.000	−2.492	0.063

p-values are exact two-tailed significance values from the Mann–Whitney U test, rounded to three decimal places.

The experimental group reported higher satisfaction than the control group in three dimensions: improvement in operational ability (*p* = 0.023), instructor enthusiasm (*p* = 0.007), and overall self-evaluation (*p* = 0.015). These differences were statistically significant.

No statistically significant differences were observed between the two groups in the remaining five dimensions: improvement in theoretical knowledge (*p* = 0.247), interest in learning (*p* = 0.105), self-study ability (*p* = 0.105), sense of participation (*p* = 0.063), and overall satisfaction (*p* = 0.063).

## 4 Discussion

The purpose of this study was to evaluate the effectiveness of the flipped classroom combined with TBL teaching model in the standardized training of ophthalmology residents, using pterygium excision surgery as the operative task. Through a comprehensive comparative analysis of before and after training theoretical scores, after training skill assessment results, and questionnaire survey data between the two groups, we found that the experimental group achieved significantly higher theoretical scores than the control group, with a large effect size (Cohen's *d* = 0.95). In the practical skill assessment, the total score of the experimental group and the effect sizes for multiple subdomains reached a very large magnitude (Cohen's *d* up to 5.29). These differences highlight the unique value of this teaching model in ophthalmic surgical skills training.

However, the questionnaire results revealed a somewhat contradictory pattern: residents in the experimental group reported significantly higher ratings than the control group in terms of self-perceived improvement in operational ability, teacher enthusiasm, and overall self-evaluation, whereas no statistically significant differences were observed in self-assessed theoretical knowledge consolidation, learning interest, sense of participation, or overall satisfaction with the teaching process. These seemingly scattered and even contradictory findings provide rich material for an in-depth understanding of the internal structure and applicability boundaries of the flipped classroom combined with TBL model. Below, we discuss each aspect in turn.

To begin with, among the five modules of the after training skill assessment, ranked by effect size, the order was: operative procedure, post-operative handling, operational proficiency, question answering, and pre-operative preparation. Among these, the first three demonstrated very large effect sizes. This indicates that the flipped classroom combined with TBL model significantly enhances learners' surgical skills. In addition to the fact that this model ensures more hands-on practice time and opportunities in the classroom (26), we propose two further explanations.

First, the difficulty of training residents in pterygium excision surgery lies in the need to perform multi-step fine manipulations under a microscope (27). Traditional teaching methods are more likely to cause cognitive overload in learners. According to cognitive load theory (CLT) (28), the objective complexity of this surgical task results in a high intrinsic cognitive load. The traditional teaching model, where instructor-led dense theoretical lectures and guided skill practice occur within the same class session, significantly increases the extraneous load. Moreover, the germane load—the cognitive effort required to transform theoretical knowledge into action strategies—is also difficult to accomplish within a single class period (29). It is now widely recognized in education that excessive cognitive load can be detrimental to skill acquisition (30). Therefore, instructional design for ophthalmic surgical skills training should aim to reduce extraneous load and decompose intrinsic load to maximize teaching effectiveness. The flipped classroom restructures the traditional teaching sequence by moving knowledge acquisition to the pre-class phase. Learners can repeatedly watch standardized surgical videos, encoding the procedural steps into long-term memory in advance, and then focus on hands-on practice during class. By breaking down the learning task, this approach substantially reduces the intrinsic load of skill training. Furthermore, the flipped classroom combined with TBL is learner-centered. TBL, in particular, shifts instructor-directed teaching toward peer interaction and feedback (31). Team members, being at roughly the same skill level, can better understand each other's learning progress and needs, which effectively reduces the pressure of trial-and-error during practice (32). Thus, the new teaching model further lowers the extraneous load. Under the dual mechanism of “time restructuring + team collaboration,” residents in the experimental group were able to devote more cognitive resources to proceduralizing the motor skills.

Second, the teaching process included within-group role rotation, through which learners internalized knowledge from three different perspectives: as the operator, as the assistant, and as the observer. As the operator, they explained the procedure; as the assistant, they asked questions; as the observer, they recorded and provided feedback. These three roles allowed key steps to be repeatedly addressed, and peer experience sharing helped to supplement easily overlooked tacit steps. This may explain why the experimental group achieved a notably higher scoring rate and a very large effect size in the post-operative handling module—a component often weakened as mere “clean-up work” and not given sufficient attention in traditional teaching. Therefore, we believe that the flipped classroom combined with TBL model facilitates the internalization of knowledge from lower levels (memorization, comprehension) to higher levels (application, analysis, evaluation) (33), thereby laying a solid foundation for future creative practice.

In contrast to the overwhelming superiority in post-training practical skills, the experimental group's post-training theoretical examination scores, although significantly higher than those of the control group, showed a much smaller effect size (*d* = 0.95). Moreover, in the questionnaire survey, the between-group difference in satisfaction with whether the teaching model consolidated and improved theoretical knowledge did not reach statistical significance. This phenomenon warrants reflection on the alignment between theoretical knowledge assessment and teaching objectives in surgical skills training.

In the pre-class theoretical knowledge self-study module of the experimental group, the instructor set learning objectives that required residents to explain the procedure and answer questions during class. Consequently, residents in the experimental group may have focused more on procedural steps and precautions than on a broader range of theoretical knowledge. The theoretical examination used in this study consisted of type A and type B questions. Type A questions were oriented toward anatomical knowledge, surgical contraindications, and similar declarative knowledge—content that could also be mastered through traditional teaching methods. Furthermore, in traditional teaching, the instructor provides systematic and focused explanations, whereas less experienced junior residents in the experimental group might not have been able to cover all knowledge points during self-study. This may also explain why the “self-study ability” dimension in the questionnaire showed no significant between-group difference, indicating that residents in the experimental group subjectively did not feel that they had memorized more knowledge points.

These findings offer the following implications for future research: first, the proportion of application-oriented objective questions in theoretical knowledge assessments should be increased; second, during the pre-class self-study phase, instructors could provide learners with an outline of key theoretical points to help them focus their learning.

Another noteworthy finding from the questionnaire survey is that the experimental group rated “instructor enthusiasm” significantly higher than the control group. In traditional lecture-based teaching, the instructor acts as a one-way transmitter of knowledge, and teaching enthusiasm is often constrained by fixed teaching tasks (34). In the new model, however, the instructor's role shifts from transmitter to guide, observer, and feedback provider. Moreover, the enhanced interactivity of TBL may stimulate instructors' teaching interest. When instructors become genuinely involved in students' skill acquisition, their teaching enthusiasm is maximally awakened and activated, which in turn strengthens the classroom learning climate and fosters a positive interaction loop between instructors and students (35).

Nevertheless, the questionnaire also showed that the experimental group did not differ significantly from the control group in “learning interest,” “sense of participation,” or “overall satisfaction.” These results deserve further exploration. Upon reviewing the individual questionnaire scores, we found that all residents in the experimental group gave the highest possible scores in these dimensions, indicating that they generally perceived the new teaching method as enhancing their learning interest, participation, and satisfaction. The lack of significant differences compared to the control group may be because pterygium excision surgery is inherently an important and technically demanding procedure in ophthalmology; subjectively, both groups of residents had a strong intrinsic interest in this skill training—a motivation that can substantially enhance learning efficiency (36).The absence of statistically significant differences in learning interest, sense of participation, and overall satisfaction should be interpreted with caution. In the experimental group, the median scores for these items reached the maximum (4.0), suggesting a ceiling effect. The lack of significance is therefore more likely attributable to limited statistical power and ceiling constraints than to the absence of a true educational benefit. Larger sample sizes and more differentiated response scales are needed in future studies to address this issue.

Overall, the flipped classroom combined with TBL teaching method significantly improves the surgical skills of ophthalmology residents. It not only teaches students how to perform procedures but, more importantly, cultivates their clinical decision-making and reflective abilities (37), which aligns well with the core goal of standardized residency training shifting toward a competency-based orientation.

Nevertheless, this study has several limitations. First, the sample size was small. Although effect sizes and non-parametric tests enhanced internal validity, external validity remains limited, and the statistical power for some questionnaire items may be affected. Future studies should expand the sample size for validation. Second, this was a single-center, single-discipline study. The teaching effectiveness of this model in other centers or disciplines requires further investigation with broader populations. Third, the skill assessment used *ex vivo* porcine eyes rather than real patient surgeries. Although this ensured standardization, the transferability of skills to real surgical settings remains to be validated. Fourth, only immediate post-training outcomes were assessed, without long-term follow-up. The decay curve for surgical skills typically begins to decline 3–6 months after training (38). Whether the skill memory established by the flipped classroom combined with TBL method is more durable than that from traditional teaching is a question that future research must address. To address the third and fourth limitations, our research team has developed a proctoring plan for the participants: an experienced senior instructor will lead each resident through a live pterygium excision surgery, with the entire procedure video-recorded. The instructor will then evaluate the resident's performance based on the video recording, allowing a more comprehensive assessment of the long-term skill retention and real-world applicability of the flipped classroom combined with TBL method. This plan involves real patients and is currently under ethical review. Now artificial intelligence and virtual reality technologies have gradually been integrated into the clinical practices (39–42) and the clinical education in ophthalmology (43, 44). How to combine new technologies with traditional teaching methods and explore a new integrated teaching and training model is also the direction of our research.

## 5 Conclusion

This study confirms that the flipped classroom combined with TBL significantly enhances the surgical skills of ophthalmology residents, demonstrating teaching outcomes markedly superior to those of traditional methods. The underlying mechanism involves optimizing cognitive load distribution and promoting team collaboration, which substantially improves residents' operative proficiency and clinical decision-making. Through feedback-based evaluation, the model refines action procedures, resulting in outstanding teaching effectiveness. Therefore, this innovative teaching approach holds high application value in standardized surgical skills training for ophthalmology residents. The model can be extended to various challenging surgical skills training programs in the future, provided that theoretical assessment content is optimized, residents' learning burden is reasonably monitored, and long-term follow-up is conducted to verify skill retention.

## Data Availability

The raw data supporting the conclusions of this article will be made available by the authors, without undue reservation.
